# Potential of entomopathogenic nematodes against the pupal stage of the apple maggot *Rhagoletis pomonella* (Walsh) (Diptera: Tephritidae)

**DOI:** 10.21307/jofnem-2020-079

**Published:** 2020-07-28

**Authors:** Muhammad Usman, Sehrish Gulzar, Waqas Wakil, Jaime C. Piñero, Tracy C. Leskey, Laura J. Nixon, Camila Oliveira-Hofman, Shaohui Wu, David Shapiro-Ilan

**Affiliations:** 1 Department of Entomology, University of Agriculture Faisalabad, Punjab, Pakistan; 2 Stockbridge School of Agriculture, University of Massachusetts, Amherst, MA; 3 USDA-ARS, Kearneysville, WV; 4 USDA-ARS, SE Fruit and Tree Nut Research Laboratory, Byron, GA; 5 Department of Entomology, University of Georgia, Tifton, GA

**Keywords:** Entomopathogenic nematode, Pupae, *Rhagoletis pomonella*, Species, Virulence.

## Abstract

The apple maggot, *Rhagoletis pomonella* (Walsh) (Diptera: Tephritidae), is considered a key pest of apples and is native to the eastern United States. The virulence of seven different species of entomopathogenic nematodes (EPN) was assessed against pupae of *R. pomonella* under laboratory conditions. Nematode species and strains included *Steinernema carpocapsae* (ALL strain), *Steinernema feltiae* (SN strain), *Steinernema riobrave* (355 strain), *Steinernema glaseri* (VS strain), *Heterorhabditis bacteriophora* (VS strain), *Heterorhabditis indica* (HOM1 strain), and *Heterorhabditis megidis* (UK211 strain). We conducted three bioassays: (i) short-term exposure cup bioassay (7 d), (ii) long-term cup bioassay (30 d), and (iii) pot bioassay (30 d). In the short-term exposure bioassay, all nematode strains (applied at 54 infective juvenile nematodes (IJs) cm^−2^) significantly reduced (range: 42.9-73.8%) insect survival relative to the control, but no differences were observed among the treatments. For the long-term exposure bioassay, using the same EPN application rate as the short exposure assay, all treatments reduced adult *R. pomonella* emergence compared with the control. *Steinernema riobrave* was the most virulent (28.3% survival), and *S. glaseri* and *H. megidis* were the least virulent (53.3% survival). In the pot experiment, *S. riobrave* and *S. carpocapsae* (applied at 27 IJs cm^−2^) had the highest virulence (23.3 and 31.7% survival of *R. pomonella*, respectively), while *H. bacteriophora* was the least effective (68.33% survival). Our results indicate that *S. riobrave*, *S. carpocapsae*, and *S. feltiae* have substantial potential to attack *R. pomonella* pupae, and their field application under the tree canopy (prior to adult emergence) in the spring when temperatures are conducive might be a good option for successful IPM of apple maggot fly.

Various fruit fly species (Diptera: Tephritidae) are serious pests worldwide that reduce the quality of mature fruits in commercial orchards (Weems and Heppner, 2014). Among them the apple maggot, *Rhagoletis pomonella* (Walsh) (Diptera: Tephritidae), is recognized as a quarantine pest of various important commercial fruit crops, i.e. apples, pears, apricots, plums, hawthorns, and crabapples. In commercial apple orchards, along with quarantine costs, *R. pomonella* induces losses in the form of reduced fruit quality and yield, and increased costs associated with pest management. If the pest remains untreated, it can reduce yield up to 70% in severe infestations (Prokopy et al., 1990; Howitt, 1993; Wise et al., 2009; Prengaman, 2018).

*Rhagoletis pomonella* has one generation per year throughout its range (Dean and Chapman, 1973). Mature larvae exit the fruit, drop to the ground, and overwinter as pupae in the soil at 2 to 5 cm depth under the infested tree. The pupae overwinter in a stage of facultative diapause that is regulated by environmental factors (Prokopy, 1968). Adults emerge from puparia beneath infested abandoned or insufficiently managed host trees and immigrate into nearby commercial apple orchards, where they oviposit into fruit flesh (Boller and Prokopy, 1976; Reissig, 1979).

To achieve commercially acceptable levels of control, apple growers typically apply up to three broad-spectrum insecticide sprays to the entire orchard, beginning in early July and ending in late August (Prokopy et al., 1990; Bostanian et al., 1999). One alternative management tactic that reduces reliance on the widespread application of broad-spectrum insecticides was developed in the form of an attract-and-kill system involving red spheres coated with Tangletrap in association with an attractive lure (Prokopy, 1968; Bostanian et al., 1999; Wright et al., 2012). Low-maintenance spheres combined with attractive visual cues, a toxicant, and a phagostimulant such as sucrose have provided effective control of *R. pomonella* in commercial apple orchards (Green and Wright, 2009; Wright et al., 2012; Morrison et al., 2016). All these management strategies target the aboveground adult stage of *R. pomonella* rather than the soil dwelling stages of *R. pomonella*.

Chemical insecticides may be effective but there are a variety of problems associated with the use of broad-spectrum chemical insecticides such as the development of pesticide resistance, resurgence, hazards to humans and the environment (Coppel and Mertins, 1997). Therefore, the development of biologically based alternatives to control fruit flies is warranted (Spinner et al., 2011). In terms of natural enemies, several species of parasitoids have been found to attack the *R. pomonella* but they are not effective under commercial orchard situations (Feder, 1995). AliNiazee (1985) evaluated two parasitoids *Opius lectoides* Gahan and *O. downesi* against *R. pomonella* in apple and found only 2% parasitism rate compared to *Rhagoletis zephyria* Snow for which 60% parasitism was observed in hawthorn fruit. Entomopathogenic nematodes may be another alternative approach for the biological control of *R. pomonella*.

Entomopathogenic nematodes (EPNs) from genera *Steinernema* and *Heterorhabditis* have the ability to infect and kill insect pests and they are naturally found in all types of agricultural and natural soils (Grewal et al., 2005). Entomopathogenic nematodes are associated with symbiotic bacteria, i.e. *Xenorhabdus* spp. bacteria are associated with *Steinernema* spp. and *Photorhabdus* spp. are associated with *Heterorhabditis* spp. Entomopathogenic nematodes enter the host via the mouth and anus (Salame and Glazer, 2000), spiracle (Triggiani and Poinar, 1976), and directly through the softer portion of insect integument (Koppenhofer et al., 2000). Upon entrance into the host, infective juvenile nematodes (IJs) release their associated bacteria; host death occurs within 24 to 48 hr due to septicemia or toxemia (Shapiro-Ilan et al., 2018). EPNs are extensively used to combat root feeding insects (Johnson and Murray, 2008; Shapiro-Ilan et al., 2017).

Numerous studies have been conducted to measure EPN efficacy against tephritids including *Rhagoletis indifferens* Curran (Stark and Lacey, 1999; Yee and Lacey, 2003), *Rhagoletis cerasi* L. (Kepenekci et al., 2015), *Ceratitis capitata* (Wiedemann) (Karagoz et al., 2009; Malan and Manrakhan, 2009; Rohde et al., 2010), several *Anastrepha* species (Toledo et al., 2005, 2006, 2009; Barbosa-Negrisoli et al., 2009; Heve et al., 2017, 2018) as well as *Bactrocera dorsalis* (Hendel) (Lindegren and Vail, 1986), *Bactrocera oleae* (Rossi) (Sirjani et al., 2009; Langford et al., 2014), and *Drosophila suzukii* (Matsumura) (Diptera: Drosophilidae) (Cuthbertson et al., 2014; Garriga et al., 2018, 2020). To the best of our knowledge, no previous research has been conducted on the efficacy of EPNs against *R*. *pomonella*. The objective of this study was to quantify the susceptibility of *R*. *pomonella* pupae to different species of EPNs. Our focus was on the pupal stage, because among the soil dwelling stages of *R. pomonella*, the last instar larval stage is very short in tephritids compared with the pupal stage (Prokopy, 1968; Meck et al., 2008) which for *R. pomonella r*emains in the soil for up to eight months depending on location before emerging as adults (Prokopy, 1968).

## Materials and methods

### Entomopathogenic nematodes

The nematodes used in this study were from the USDA International Culture Collection held in Byron, Georgia, USA. The seven EPNs species tested were *Steinernema carpocapsae* (ALL strain), *Steinernema riobrave* (355 strain), *Steinernema feltiae* (SN strain), S*teinernema glaseri* (VS strain), *Heterorhabditis bacteriophora* (VS strain), *Heterorhabditis indica* (HOM1 strain), and *Heterorhabditis megidis* (UK211 strain). The nematodes were cultured in vivo on the last instar of *Galleria mellonella* L. (Lepidoptera: Pyralidae) and IJs were collected using the White trap method (Shapiro-Ilan et al., 2016). *G. mellonella* larvae were obtained from Vanderhorst Wholesale, Inc. (St. Mary’s, Ohio). The EPNs were stored in aqueous suspensions in 250 ml tissue culture flasks at 14°C. Nematodes were stored for less than two weeks before using them in experiments.

### *Rhagoletis pomonella* colony

Pupae of *R. pomonella* from a non-diapausing strain were supplied by United States Department of Agriculture, Agriculture Research Service (USDA-ARS), Kearneysville, West Virginia. Briefly, mated females kept in a colony room at 16:8 L:D and 25°C were provided organic ‘Red Delicious’ apples as an oviposition substrate. Apples were exposed to adults for 3 to 4 days, then removed and suspended over trays of moistened sand for four weeks at 16:8 L:D, 24°C and 45% RH. Pupae were removed from sand using water and agitation to float them to the surface of trays. Removed pupae were placed in small plastic cups with tissue to absorb excess moisture and shipped overnight within two days of removal from sand.

### Short-term exposure bioassay

This experiment as well as the others described below were organized as completely randomized designs (CRD) and conducted at USDA-ARS research station in Byron, GA. Bioassay procedures were based on prior EPN virulence assays (Shapiro-Ilan et al., 2002). The experimental arena consisted of a lidded 30 ml plastic cup filled with 10 g of autoclaved sand with 0% soil moisture content. Each cup contained one pupa (recently pupated) at the bottom of the cup underneath the sand. In total, 1 ml of each nematode suspension containing 500 IJs (54 IJs cm^−2^) was applied to the top of sand via pipette, resulting in a final moisture content of 10% in each cup. The control group received the same amount of distilled water without IJs. Thus, the experiment had eight treatments consisting of seven nematode species and one negative control treatment. Each treatment was replicated three times. Lidded cups were placed on trays and bagged with damp paper towels to help retain moisture, and incubated at 25°C. Seven days after treatment application, pupae were examined under a stereomicroscope for infection by the presence of nematode inside the pupae. The experiment was conducted twice, resulting in a total of six replicates per treatment and each replicate had seven cups with (so a total of 42 pupae per treatment).

### Long-term exposure bioassay

The experimental approach was identical to the short exposure bioassay except for its duration. After inoculating nematodes to the cups and placing them in the incubator, we observed the cups daily for adult emergence until 30 days post treatment application. Adults that successfully emerged were considered to have survived the nematode treatment. All other experimental parameters were the same as described above except we used 10 pupae per replicate (based on the availability of insects).

### Pot bioassay

Five nematode species, *S. riobrave*, *S. carpocapsae*, *S. feltiae*, *H. indica*, and *H. bacteriophora* were selected for the pot assay based on their virulence performance in the prior experiments (*S. glaseri* and *H. megidis* were eliminated). Plastic pots (10.16 cm diameter) were filled with non-sterile oven-dried soil (200 g) (0% moisture). The soil was a loamy sand (84% sand, 10% silt, 6% clay; 2.8% organic matter; pH 6.1). Approximately, 27 ml of distilled water was added and mixed manually for equal distribution of water throughout the soil. After mixing, 10 pupae were loosely buried inside the soil (3 cm deep). Each nematode species was applied at 1,377 IJs ml^−1^, which is equivalent to 27 IJs cm^−2^ to the top of soil throughout the pot (with final moisture content 14%). The negative control received the same amount of distilled water (1 ml) without IJs. A 100-mm Petri dish cover lined with yellow sticky trap was placed on the top of each pot. Pots were put onto a plastic tray and bagged with damp paper towel to help retain moisture and incubated at 25°C. We observed the pots daily and monitored adult emergence until 30 days post treatment. Successfully emerging adults were considered as survived individuals. There were three replicate pots per treatment and control. The experiment was conducted twice in time.

### Statistical analysis

Treatment effects were analyzed with analysis of variance (ANOVA). If the ANOVA detected a significant difference (*P* ≤ 0.05), then treatment differences were elucidated through Tukey’s test (SAS Version 9.4, 2002). Data from repeated experiments (trials) were combined for analysis when the treatment×trial interaction was not significant. Based on the inspection of residual plots, percentage data were arcsine transformed prior to analysis (Southwood, 1978; Steel and Torrie, 1980; SAS, 2002). Non-transformed means are presented in the Results section and associated figures.

## Results

### Short-term exposure bioassay

**Figure 1:**
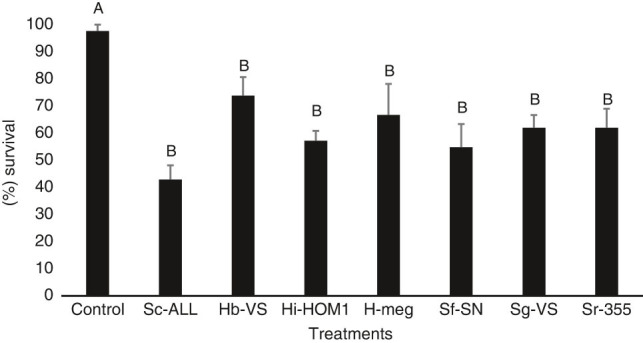
Mean percentage survival (± SEM) of *Rhagoletis pomonella* pupae when exposed to various treatments in 30-ml soil cups for 7 days (short-term bioassay). Control = no nematodes, Sc-ALL = *Steinernema carpocapsae* ALL strain, Hb-VS = *Heterorhabditis bacteriophora* VS strain, Hi-HOM1 = *Heterorhabditis indica* HOM1 strain, Hmeg = *Heterorhabditis megidis* UK211 strain, Sf-SN = *Steinernema feltiae* SN strain, Sg-VS = *Steinernema glaseri* VS strain, Sr-355 = *Steinernema riobrave* 355 strain. Different letters above bars indicate statistical significance according to ANOVA and Tukey’s test (*α* = 0.05).

In the short exposure bioassay, no significant interaction (*P* = 0.8427) was detected between the treatment and trial effects, and so data from the two trials were combined. All seven nematode species significantly reduced pupal survival compared with the control group (*F* = 3.87; df = 15.47; *P* = 0.0006, Fig. 1). The nematode treatments reduced pupal survival at a similar level, with no significant differences detected among them. Pupal survival among the treatments ranged from 42.85% (for *S. carpocapsae*) to 73.8% (for *H. bacteriophora*) (Fig. 1).

### Long-term exposure bioassay

**Figure 2:**
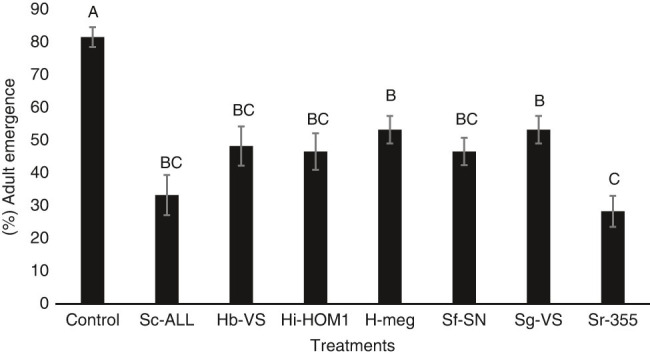
Mean percentage survival (± SEM) of *Rhagoletis pomonella* pupae when exposed to various treatments in 30-ml soil cups for 30 days (long-term bioassay). Control=no nematodes, Sc-ALL = *Steinernema carpocapsae* ALL strain, Hb-VS = *Heterorhabditis bacteriophora* VS strain, Hi-HOM1 = *Heterorhabditis indica* HOM1 strain, Hmeg = *Heterorhabditis megidis* UK211 strain, Sf-SN = *Steinernema feltiae* SN strain, Sg-VS = *Steinernema glaseri* VS strain, Sr-355 = *Steinernema riobrave* 355 strain. Different letters above bars indicate statistical significance according to ANOVA and Tukey’s test (*α* = 0.05).

In the long exposure bioassay, no significant interaction (*P* = 0.9618) was detected between the treatment and trial effects and so data from the two trials were combined. Similar to the short exposure assays, all nematode species induced lower adult emergence compared with the control (*F* = 4.68; df = 15.47; *P* = 0.0001, Fig. 2). *Steinernema riobrave* was the most virulent EPN, resulting in the lowest adult emergence (28.3%), which was significantly lower than those treated with *S. glaseri* and *H. megidis* (53.3%). *Steinernema carpocapsae*, *S. feltiae*, *H. indica*, and *H. bacteriophora* had intermediate effects, which were not significantly different from any treatment other than control (Fig. 2).

### Pot experiment

**Figure 3:**
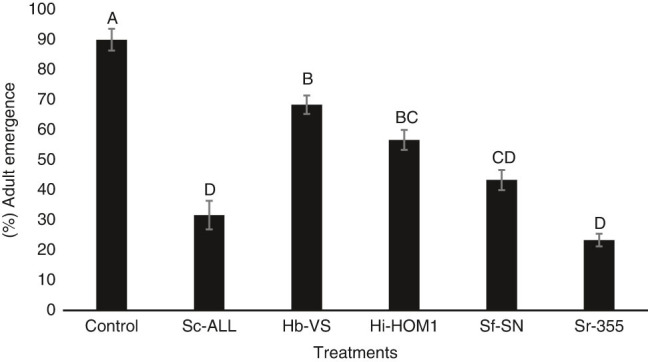
Mean percentage adult *Rhagoletis pomonella* emergence (± SEM) following exposure to various treatments for 30 days in a pot experiment. Control=no nematodes, Sc-ALL = *Steinernema carpocapsae* ALL strain, Hb-VS = *Heterorhabditis bacteriophora* VS strain, Hi-HOM1 = *Heterorhabditis indica* HOM1 strain, Sf-SN = *Steinernema feltiae* SN strain, Sr-355 = *Steinernema riobrave* 355 strain. Different letters above bars indicate statistical significance according to ANOVA and Tukey’s test (*α* = 0.05).

No significant interaction (*P* = 0.6224) was detected between the treatment and trial effects and so data from the two trials were combined. All five EPN species significantly reduced *R. pomonella* adult emergence (23.33-68.33%) compared with the control (90.0%) (*F* = 15.87; df = 11.35; *P* = 0.0001, Fig. 3). Numerically, the lowest *R. pomonella* adult emergence was observed in *S. riobrave* (23.3%) and *S. carpocapsae*. In contrast, the maximum adult emergence was recorded in *H. bacteriophora* (68.3%) and *H. indica* (56.7%) (Fig. 3). *Steinernema riobrave* and *S. carpocapsae* had similarly high levels of virulence, which were significantly stronger (with lower adult emergence) than *H. indica* and *H. bacteriophora* but not different from *S. feltiae*. *S. feltiae* caused lower adult emergence than *H. bacteriophora* only (Fig. 3).

## Discussion

In the current study, we tested seven species of entomopathogenic nematodes against pupae of *R. pomonella* in a short-term and long-term exposure assays and also in a soil pot experiment. Although no differences in nematode virulence were observed in the short-term assay, the experiment demonstrated that all EPN species are virulent to the pupae even when the duration of exposure is relatively short. When pupae were exposed to the nematodes for a longer period, all nematodes were separated from the control but *S. riobrave* was the only one that separated from other species showing higher virulence than two of the heterorhabditids. In the pot test, *S. riobrave* and *S. carpocapsae* were more effective than other treatments (though not statistically separated from *S. feltiae*). Thus, we conclude that *S. riobrave, S. carpocapsae*, and *S. feltiae* show the greatest promise for control of *R. pomonella.*

Our findings are in agreement with prior studies that indicate the tested EPN species are pathogenic to various fruit fly species. For example, *S. carpocapsae* has been shown to be virulent to *R. indifferens*, *Aanstrepha ludens* Loew*, Anastrepha obliqua* (Macquart), *B*. *oleae, C*. *capitata*, *Dacus ciliatus* Loew*, Bactrocera tryoni* Froggatt, *A. suspensa* Loew (Yee and Lacey, 2003; Lezama-Gutiérrez et al., 2006; Karagoz et al., 2009; Sirjani et al., 2009; Toledo et al., 2009; Kamali et al., 2013; Langford et al., 2014; Heve et al., 2017). In turn, *S. feltiae* has been shown to be effective against *R. indifferens*, *B. oleae*, *A. fraterculus* (Wied.), *C. capitata*, *B*. *tryoni*, *R. cerasi*, *A. suspensa* (Yee and Lacey, 2003; Barbosa-Negrisoli et al., 2009; Karagoz et al., 2009; Sirjani et al., 2009; Langford et al., 2014; Kepenekci et al., 2015; Heve et al., 2017). One of the top performers in the present study, *S. riobrave,* caused mortality in *A. ludens*, *B. oleae*, and *A. fraterculus* (Lezama-Gutiérrez et al., 2006; Barbosa-Negrisoli et al., 2009; Sirjani et al., 2009). The other species that were tested also have been reported to cause mortality in various fruit fly species (Toledo et al., 2006; Sirjani et al., 2009; Heve et al., 2017).

In the pot experiment we used the non-sterile soil, which is similar to soil that would occur under field conditions. Under these conditions, we observed substantial virulence of EPNs, especially *S. riobrave* and *S. carpocapsae*, which caused the lowest survival of *R. pomonella*. During a preliminary pot bioassay, we compared *R. pomonella* emergence among three EPN species and a control in sterile vs. non-sterile soil (four replicates each) and we did not detect any differences in EPN virulence (Usman et al., unpublished data). Moreover, in the pot experiment we placed pupae at a depth of 3 cm, which mimics the depth of pupae found under field conditions (https://apples.extension.org/apple-maggot/; Hulthen and Clarke, 2006). Therefore, we speculate that the virulence observed in our bioassays may be predictive of what we may observe in the field. Nonetheless, we realize some other biotic and abiotic factors that were not present in our study would be present in an agricultural setting (Shapiro-Ilan et al., 2017, 2018).

Similar to our study, there are various reports of pupal susceptibility among fruit flies exposed to EPN species (Stark and Lacey, 1999; Hernández, 2003; Barbosa-Negrisoli et al., 2009; Cuthbertson and Audsley, 2016; Heve et al., 2017). For example, Stark and Lacey (1999) reported that *S. carpocapsae* (72.5 and 83.3%), *S. feltiae* (70.3 and 73.3%), *H. bacteriophora* (62.5 and 60.0%), and *S. riobrave* (40.0 and 40.3%) caused pupal mortality in two experiments when *R. indifferens* was exposed during the larval stage. Also, in agreement with our study, *S. riobrave* was reported to cause high levels of pupal mortality in another fruit fly *A. fraterculus* (Barbosa-Negrisoli et al., 2009). In contrast, other studies did not observe pupal infection among different species of fruit flies when exposed to various EPNs strains (Yee and Lacey, 2003; Malan and Manrakhan, 2009; Langford et al., 2014; Garriga et al., 2020). For example, Yee and Lacey (2003) did not observe any pupal mortality in *R. indifferens* when treated with *S. carpocapsae, S. interdium*, and *S. feltiae.* The lack of infection in Yee and Lacey’s (2003) study may have been due to age of pupae used; they were later in development compared to Stark and Lacey (1999) study. We conducted our experiments on pupae from a continuous, non-diapausing colony. Thus, additional research is needed to verify that similar results would be obtained in wild populations.

In this study, we selected pupae of *R. pomonella* as the target stage, because the pupae undergo longer time in soil compared to the larval stage and thus are a more realistic option in practical applications. In this study we used a non-diapausing strain so additional research may be required to determine if any differences in EPN virulence exist with other wild strains. Late third-instar tephritid larvae only last for a few hours depending upon the species before pupating (Özdem and Kılınçer, 2008; Sirjani et al., 2009; Kamali et al., 2013). Under field conditions, to infect the larvae, EPNs must be present in the soil before the larvae drop from fruit or at least close to that timing (thus there is a narrow window of exposure). A number of studies indicated that when targeting the larval stage fly mortality actually occurred during pupal stage due to very short duration of late third instars (Lindegren and Vail, 1986; Karagoz et al., 2009; Sirjani et al., 2009; Kamali et al., 2013; Heve et al., 2017). Also, another benefit of using pupae as the target stage is that *R. pomonella* overwinters in the soil providing more exposure time to EPNs. EPNs can potentially be applied to the soil in the spring when pupae are present and soil temperatures are conducive to infection (Shapiro-Ilan et al., 2017).

Entomopathogenic nematodes enter the host via mouth and anal opening (Salame and Glazer, 2000), spiracle (Triggiani and Poinar, 1976), and directly through the softer portion of insect integument (Koppenhofer et al., 2000). Several studies have described possible mechanisms of infection in the pupal stage. Fraenkel and Bhaskaran, (1973) observed EPNs to be pathogenic to pupae due to their entrance via intersegmental membranes prior to completion of cuticle development. In addition, according to Barbosa-Negrisoli et al. (2009), soft portion of integument might be responsible for *A. fraterculus* pupal susceptibility. Along these lines, we exposed early stage pupae to EPNs in our study, which probably had a relatively soft integument. In another study, the mouth, anus, and spiracles were found to be still open during the pupal stage and hence offer several options for EPN entry routes (Minas et al., 2016). Heve et al. (2017) stated that larger and longer steinernematids such as *Steinernema diaprepesi*, *S. glaseri* and others in the *S. glaseri* group are more virulent to pupae of *A. suspensa* due to the presence of more fat reserves in the nematode that allow for prolonged exposure to the pupae. However, this premise is not supported in our results because the larger nematodes (*S. glaseri, H. megidis,* and *S. feltiae*) were not the most virulent.

Entomopathogenic nematodes exhibit different foraging behaviors that lie on a continuum between ambushers and cruisers (Grewal et al., 1994; Lewis et al., 2006). Ambush foraging nematodes tend to remain near the soil surface, nictate, and attack passing insects. Cruiser behavior consists of active searching for the host in response to various volatile cues. A number of EPNs display foraging strategies that are intermediate between ambush and cruiser types. The nematodes that we observed to be the most virulent to *R. pomonella* can be classified as ambusher (*S. carpocapsae*) or intermediate foragers (*S. riobrave* and *S. feltiae*) (Lewis et al., 2006). The cruiser-type EPNs that we tested (the heterorhabditids and *S. glaseri*) did not perform as well. Nonetheless, it is not clear that foraging strategy played a major role in causing differential virulence in our study. In our relatively small arenas, foraging behavior may not have been critical to successful infection; field testing will be needed to confirm any trends in EPN foraging behavior as a factor in biocontrol efficacy.

Our results indicate that EPNs such as *S. riobrave*, *S. carpocapsae*, and *S. feltiae* have significant potential to suppress *R. pomonella* pupal populations. A possible approach would be to apply the nematodes under the tree canopy in spring when the soil temperature is conducive, before pupae emerge into adults in the summer. *Rhagoletis pomonella* normally pupate in the fall and remain inside the soil until late June or July, thus providing sufficient time for EPNs to infect. Expanding on the soil application concept, it may be possible develop a trap-tree approach using EPNs against *R. pomonella* similar to what has been developed for the plum curculio, *Conotrachelus nenuphar* (Herbst.) (Coleoptera: Curculionidae). This novel attract-and-kill approach calls for baiting the branches of several perimeter-row trees with a synergistic lure, which results in aggregations of adult *C. nenuphar* on those trap trees, and then confining insecticide applications to those trees only (Leskey et al., 2008). EPNs are then applied to the soil underneath the canopies of trap trees, which are known to concentrate injury by *C. nenuphar*, to control resulting ground-dwelling stages (Leskey et al., 2008; Shapiro-Ilan et al., 2013). Before attempting such field applications, the impact of climate conditions such as temperature and soil moisture on EPN efficacy and post-application persistence should be evaluated so that the optimum conditions for infection are identified (Shapiro et al., 1999, 2000). Additional research will be required to assess the efficacy of EPNs under semi-field pot experiments and field cages so that a successful IPM program for *R. pomonella* can be developed.
